# Osteoarthritis knee and modifiable cardiovascular risk factors: play in tandem

**DOI:** 10.12669/pjms.39.6.7596

**Published:** 2023

**Authors:** Abrar Ahmed Wagan, Paras Surahyo, Abdul Qadir Bhutto, Ammad Asghar

**Affiliations:** 1 Abrar Ahmed Wagan, MBBS, FCPS, FCPS, FACR Associate Professor of Rheumatology Indus Medical College, Tando Mohammad Khan; 2 Paras Surahyo, MBBS, FCPS Assistant Professor, Department of Radiology, Bilawal Medical College, Jamshoro, Pakistan; 3 Abdul Qadir Bhutto, MBBS, MD Assistant Professor, Department of Cardiology Pir Abdul Qadir Shah Jilani Medical College Gambat; 4 Ammad Asghar, MBBS, FCPS, FCPS Assistant Professor of Medicine, Department of Medicine Sahiwal Medical College Sahiwal

**Keywords:** Knee OA, CVD risk factors, Metabolic syndrome, Hypercholestrolemia, LDL

## Abstract

**Objective:**

To know the frequency of modifiable cardiovascular risk factors in knee osteoarthritis patients.

**Method:**

Cross sectional study was done at Department of Rheumatology Indus Medical College, Tando Mohammad Khan from March 25, 2022 to November 24, 2022. Total 246 Osteoarthritis of knee cases with (Kellgren-Lawrence grad-II and above) on x-ray, were selected after demographic details, blood pressure, body mass index and physical examination was done, 5ml of venous blood was drawn by phlebotomist, sent for fasting blood sugar, serum lipids analysis and Framingham 10years risk score was calculated afterward for each participant.

**Results:**

In this study males (126) and females (120). Overall (78%) had risk factors, Patients having one CVD risk factor were (22.8%), two risk factors in (21.1%), three in (21.5%), four (9.8%) and five factors in (1.6%) while frequency of modifiable cardiovascular risk shows obesity (45.5%) hypertension (40.2%) intermediate to high risk of framingham score (40%) diabetes mellitus (25%), smoking (17%), high low density lipoproteins (8.1%). In males obesity(54.2%), hypertension (47.5% ) and (45.8%) were on medication, diabetes mellitus(31.7%), smoker(31%), high risk FRS(39.2%), K-L grade-IV(58.4%) and in females: obesity (42%), hypertension (43.7%) and (40.5%) were on medication, diabetes mellitus in (19%), smoking (4%), high risk FRS (13.5%), K-L Grade-4 (42%), significant association of diabetes mellitus, smoking, FRS and K-L grades with gender (p<0.05).

**Conclusion:**

In OA knee there is high prevalence of modifiable cardiovascular risk factors and together these imposes a major health risk for future cardiac events and disability.

## INTRODUCTION

Osteoarthritis (OA) is one of the most prevalent joint problems, affects weight bearing joints of body. It is the 6^th^ leading cause of disability in the world, causes activity limitations, affects participation, quality of life, wellbeing, and risk of mortality is increased due to cardiovascular problems, directly linked with disability level. Worldwide prevalence is around 3.5%, occurs in old age and its increasing in developed and developing countries, very important public health problem.^1^,^2^

The prevalence ranges from 12.3% to 21.6% in France and USA ,more in women than men, common in developed than developing countries.^1^ According to the COPCORD study In Iran’s, rural population the prevalence was high (19.3%).^3^ OA is idiopathic but related to various risk factors, grouped into: modifiable- personal level (obesity, certain occupations and sports, joint injury, joint misalignment, quadriceps muscle weakness) and Non-modifiable joint level (age, sex, ethnicity, genetics, and previous history of injury).^4^

Australian national health survey (2017-2018) shows risk of cardio-vascular deaths is increased in (inflammatory and degenerative joint diseases), 1.5-2.0 fold rise in RA, 1.3 fold in systemic connective tissue disorders, 1.5 gout and twofold in OA, and these patients are at increased risk of having obesity, diabetes, hypertension, dyslipidemia specially in younger age.^5^ Korean study, those with knee OA had increased risks of CVD (HR) 1.26, 95% confidence interval (CI) 1.15-1.38), myocardial infarction, and stroke then normal individuals and interestingly those who did not exercise had an increased risk of CVD (HR 1.25, 95% CI 1.11-1.40), and no risk in those who exercised at least once a week (HR 1.11, 95% CI 0.96-1.28).^6^,^7^ Grade-4 knee OA patients had higher frequency of cardiovascular risk factors (p<0.05) and less life expectancy then Grade-2.^2^ Chinese and Pakistani studies has shown increased prevalence of metabolic syndrome that (58.9%) in OA patients.^8^ Research suggests key roles of meta-inflammation, (adipokines-leptin) in OA, metabolic syndrome and CVD, the role of adiponectin remains controversial in both CVD and OA as it may exert pro-inflammatory and anti-inflammatory effects.^9^

Osteoarthritis could be potential major risk factor for cardiovascular problems, secondary to difficulty in walking and exercise ability; risks can be reduced with regular walk/exercise and proper management of osteoarthritis and vice versa. Our objective was to know the frequency of modifiable cardiovascular risk factors in knee osteoarthritis patients.

## METHOD

This cross sectional study was performed at Department of Rheumatology Indus Medical College from March 25, 2022 to November 24^th^ 2022.

### Inclusion & Exclusion Criteria

To know the frequency of modifiable cardiovascular risk factors in knee osteoarthritis patients. All those patients who had history of pain in both knee joints and fulfilling American college of rheumatology criteria osteoarthritis criteria and K-L Grade-2 and above were enrolled, autoimmune diseases, (RA, SLE, Scleroderma, Psoriatic arthritis) and those who had history of surgery to their knee joint due to any cause in last one year and currently using lipid lowering medicines were excluded.

### Ethical Approval

The study was approved by the Ethics Committee of the Hospital wih Ref.(IRB No: IRB/51/2022) Tando Mohammad Khan. Written and informed consents were taken from each participant.

Demographic data like age, disease duration, smoking habits, history of blood pressure and diabetes mellitus with medications in use were asked , waist circumference, height and weight were noted for body mass index, blood pressure was measured after five minutes rest with best of two reading was noted, Radiologist did X-rays reporting on basis of Kellgreen-lawrence Grading : Grade-1: doubtful narrowing of joint space and possible osteophytes, Grade-2: definite osteophytes, definite narrowing of joint space, Grade-3: moderate multiple osteophytes, and definite narrowing of joints space, some sclerosis and possible deformity of bone contour, Grade-4: large osteophytes, marked narrowing of joint space, severe sclerosis and definite bony deformity of. Participants were instructed to come on next morning with 14 hours fasting state, 5 ml of blood was taken, samples were sent to laboratory for fasting blood sugar and if someone has level of >126mg/dl test was repeated on second day), Lipid analysis. Each individual’s 10 years FRS risk was calculated, and categorized into<10%score=low,11-20% =intermediate. >20%= high.

IBM-SPSS version 23.0 was used, Mean with standard deviation were reported on age (years), BMI, SBP, DBP, Cholesterol, TG, HDL, LDL (F), FRS and disease duration. Means of these variables were compared with respect to gender using independent sample t-test. Counts with percentages were reported on BMI level, hypertension, diabetes mellitus, smoking, FRS and K-L grades. Association of these variables with gender was tested using Pearson Chi Square test.

## RESULTS

Out of 246 patients, in 120 males,the mean of studied variables: age 51.9 (SD=±8.4) years, BMI 29.5 (SD=±4.8) kg/m2, SBP 128.6mmhg (SD=±15.2), DBP 84.3mmhg (SD=±9.9), Cholesterol 195 mg/dl (SD=±40.2) ,TG 210.8 mg/dl (SD=±91.9),HDL 45.4mg/dl (SD=±6.6), LDL (F) 105.3mg/dl(SD=±40.5),FRS 16.2 (SD=±10.6) and disease duration 6.1 (SD=±4.2) years. Females (126), mean age 49.7 (SD=±8.6) years, BMI 28.8 (SD=±5.2) kg/m2,SBP 126.8 (SD=±13.4), DBP 82.9 (SD=±8.9) , Cholesterol 181.8 (SD=±33.9), TG 201.7 (SD=±84),HDL 45.1 (SD=±7.1), LDL (F) 96.4 (SD=±34.1), FRS 8.2 (SD=±8.4) and disease duration 5.0 (SD=±4.2) years with significant mean differences for Age, cholesterol, and FRS scores to gender (p<0.05) (Table-I).

**Fig.1:**
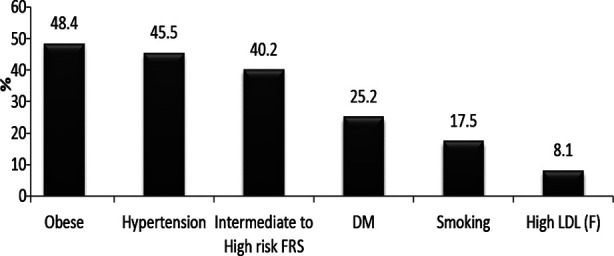
Prevalence of Modifiable cardiovascular risk factors

**Fig.2:**
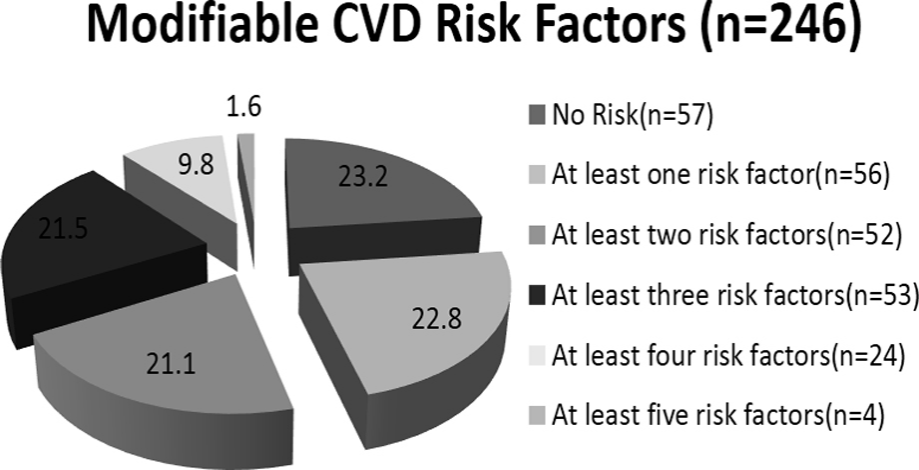
Frequency of CVD risk factors.

**Table-I:** Basic Characteristics of samples Data (n=246).

Characteristics	Male (n=120)		Female (n=126)		p-value

	Mean	SD	Mean	SD	
Age (years)	51.9	8.4	49.7	8.6	0.04*
BMI	29.5	4.8	28.8	5.2	0.29
SBP	128.6	15.2	126.8	13.4	0.32
DBP	84.3	9.9	82.9	8.9	0.22
Cholesterol	195.0	40.2	181.8	33.9	<0.01*
TG	210.8	91.9	201.7	84.0	0.41
HDL	45.4	6.6	45.1	7.1	0.77
LDL (F)	105.3	40.5	96.4	34.1	0.06
FRS	16.2	10.6	8.2	8.4	<0.01*
Disease Duration	6.1	4.2	5.0	4.2	0.058

### Comparative analysis, among males

obesity (54.2%),hypertension (47.5%) and(45.8%) were on medication, diabetes mellitus(31.7%), smoker(31%), high risk FRS(39.2%), K-L Grade-4 (58.4%).In females: obesity (42%), hypertension (43.7%) and (40.5%) were on medication, diabetes mellitus(19%), smoker (4%), high risk FRS (13.5%), K-L Grade-4 (42%), significant association of Diabetes mellitus, smoking, FRS and K-L grades with gender (p<0.05).

### Overall frequency of modifiable cardiovascular risk shows

obesity (45.5%), hypertension (40.2%), intermediate to high risk of FRS (40%),diabetes mellitus (25%), smoking (17%), high LDL (8.1%). Bar chart-1. Out of 246 patients 189 (76.8%) samples were found with modifiable CVD risk factors. One CVD risk factor in (22.8%), two (21.1%), three (21.5%), four (9.8%) and five factors (1.6%). Pie chart-1

On association of FRS and K-L Grade-4 (34%) were low risk FRS, 60% with intermediate risk FRS and 81.3% with high risk FRS, a significant association of FRS and K-L grades (p<0.05) Table-III. In modifiable CVD risk factors significant association of BMI, Hypertension, smoking, LDL (F), DM and FRS was observed with K-L grades, p<0.01 (Table-IV).

**Table-II:** Comparative Analysis of Studied variables.

Characteristics		Sex				p-value

		Male		Female		

		n	%	n	%	
BMI	<24.9 Normal	23	19.2	30	23.8	0.20
	25 - 29.9 Overweight	32	26.7	42	33.3	
	>30 Obese	65	54.2	54	42.9	
Hypertension	Yes	57	47.5	55	43.7	0.54
	No	63	52.5	71	56.3	
Hypertension with Medicine	Yes	55	45.8	51	40.5	0.39
	No	65	54.2	75	59.5	
Diabetes Mellitus	Yes	38	31.7	24	19.0	0.02*
	No	82	68.3	102	81.0	
Smoking	Yes	38	31.7	5	4.0	<0.01*
	No	82	68.3	121	96.0	
Framingham Score	Low risk	50	41.7	97	77.0	<0.01*
	Intermediate risk	23	19.2	12	9.5	
	High risk	47	39.2	17	13.5	
K-L Grade	Grade-II	10	8.3	25	19.8	<0.01*
	Grade-III	40	33.3	48	38.1	
	Grade-IV	70	58.3	53	42.1	

* p<0.05 was considered statistically significant using Pearson Chi Square test.

**Table-III:** Association of FRS with K-L grades

Risk Factors		FRAMINGHAM SCORE						p-value

		Low risk		Intermediate risk		High risk		

		n	%	n	%	n	%	
K-L GRADE	Grade-II	35	23.8	0	0.0	0	0.0	<0.01*
	Grade-III	62	42.2	14	40.0	12	18.8	
	Grade-IV	50	34.0	21	60.0	52	81.3	

* p<0.05 was considered statistically significant using Pearson Chi Square test.

**Table-IV:** Association of Modifiable Risk Factors with K-L grades.

Risk Factors		K-L Grade						p-value

		Grade-II		Grade-III		Grade-IV		

		n	%	n	%	n	%	
BMI	<24.9 Normal	18	51.4	18	20.5	17	13.8	<0.01*
	25 - 29.9 Overweight	15	42.9	26	29.5	33	26.8	
	>30 Obese	2	5.7	44	50.0	73	59.3	
Hypertension	Yes	7	20.0	30	34.1	75	61.0	<0.01*
	No	28	80.0	58	65.9	48	39.0	
Smoking	Yes	1	2.9	13	14.8	29	23.6	0.01*
	No	34	97.1	75	85.2	94	76.4	
LDL-F	Optimal	28	80.0	53	60.2	58	47.2	<0.01*
	Above Optimal	7	20.0	19	21.6	39	31.7	
	Borderline High	0	0.0	11	12.5	11	8.9	
	High	0	0.0	5	5.7	15	12.2	
Diabetes Mellitus	Yes	3	8.6	16	18.2	43	35.0	<0.01*
	No	32	91.4	72	81.8	80	65.0	
Framingham Risk	Intermediate	35	100.0	62	70.5	50	40.7	<0.01*
	High risk	0	0	14	15.9	21	17.1	

* p<0.05 was considered statistically significant using Pearson Chi Square test.

## DISCUSSION

The mechanisms between OA and increased cardiovascular events are not fully understandable; many factors play their role, first interplay between OA and most of cardiovascular risk factors, second commonly used medicines are non-steroidal anti-inflammatory drugs which enhance the risk, third physical inactivity due to severe pain and lastly, the hall mark features of CVD (arterial thickening, stiffness, and atherosclerosis), leads to ischemia and impairs bone and cartilage nutrition causing several bone infarcts, a feature of advance OA.^10^ An observational study results showed that in hypertensive, pre-diabetics and diabetics, OA knee was more prevalent than healthy persons.^11^

This study shows higher prevalence of hypertension (45.5%), diabetes mellitus (25.5%) and high framingham risk score in comparison to western world with less mean age 49-51 years. A Systematic review and meta-analysis study revealed that OA was related to a 31% increased risk of myocardial infarction.^12^ Another meta-analysis showed: five unit increase in BMI was associated with 35% more risk of knee OA (RR: 1.35; 95%CI: 1.21, 1.51) this relationship was stronger in women than in men (p=0.04).^13^ Moghimi N et al. in (COPCORD stage-I study), reported that obesity and being overweight are related with Knee OA developement.^14^

In our cohort Obesity (45%) was also more prevalent than other risk factors, which makes someone more vulnerable to OA and future cardiovascular events. Amsterdam Osteoarthritis cohort showed knee or hip OA patient had more activity limitations in association with CVD and its risk factors and poorly performed on get up-go and stair-climb test.^15^ The risk of common heart diseases is increased in OA,(Angina, congestive heart failure, myocardial infarction) in men (adjusted overall OR (95% CI)1.45 (1.36 to 1.54) ( 1.35 (1.21 to 1.50) and women (1.51 1.39 to 1.64) with more risk of myocardial infarction.^16^ Nicola V et al.in older population without CVD, found that OA at baseline will lead to incident CVD later on.^17^-^19^

In Korean nationwide health survey, OA patients had more hypertension, diabetes mellitus, lipid problems, angina and myocardial infarction than healthy population.^20^ Our study results showed that with increasing (K-L OA) grades there is increase in framingham risk score so if disease is not treated in early stage this will increases CVD risk. A Canadian population study reported that compared to Non OA patients those who had OA knee and underwent knee replacement surgery had 26% higher chance of cardiovascular diseases.^21^

Kluzek et al. reported that OA was not only associated with increased risk of cardiovascular disease, but increased the risk of all-cause and cardiovascular mortality.^22^ In Japanese (ROAD) study knee OA is associated with metabolic syndrome, similar results were seen in elderly Americans as well.^23^,^24^ As OA cases are increasing the chance developing CVD are increased and major contributor to this risk augmentation are NSAIDs (41%) used for its treatement.^25^ Our study adds great value in research on this topic as we found there is high number of treatable cardiovascular factors in OA which is very alarming, these factors and OA propagates each other depending upon disability, so timely investigations and judicious use of medicine and newer therapies are required which helps in treating both problems.

### Limitations

This is the first study which has extensively focused on modifiable CVD risk factors in local setup with Knee OA, although sample size is small and results are not true representation of entire population, but helps in understanding the implicit role of these two disorders with each other.

## CONCLUSION

In chronic bone and joint diseases like OA knee there is very high chances of having cardiovascular events due to hidden risk factors, commonly prescribed medicines and physical inactivity due to disease itself. As such it will be highly helpful to investigate and treat them accordingly and encourage patients to adopt healthy life style and daily exercise within their limitations is highly helpful.

### Authors’ Contribution:

**AAW:** Design, drafting, data acquisition data analysis, data interpretation, accuracy, final approval. He is also responsible for the integrity and accuracy of the study.

**PS AQ AA:** Data analysis and interpretation, Critical Review and final approval of the manuscript.
